# Mechanistic and Structural Analysis of Aflatoxin B1 Degradation by *Bacillus safensis* Multicopper Oxidase

**DOI:** 10.3390/foods15081451

**Published:** 2026-04-21

**Authors:** Dongwei Xiong, Jiayi Yang, Peng Li, Shuhua Yang, Miao Long

**Affiliations:** Key Laboratory of Zoonosis of Liaoning Province, College of Animal Science and Veterinary Medicine, Shenyang Agricultural University, Shenyang 110866, China; 2022200183@stu.syau.edu.cn (D.X.); 2023220648@stu.syau.edu.cn (J.Y.); lipeng2018@syau.edu.cn (P.L.); yangshuhua0001@syau.edu.cn (S.Y.)

**Keywords:** aflatoxin B1, multicopper oxidase, molecular dynamics simulation, biosafety assessment, detoxification

## Abstract

Aflatoxin B1 (AFB1) is a potent mycotoxin threatening food and feed safety. Here, we report the identification and characterization of a *Bacillus safensis*-derived multicopper oxidase (BsaMCO) capable of efficient AFB1 detoxification. Recombinant BsaMCO exhibited robust in vitro activity, achieving >78% degradation of AFB1 under 24 h incubation at 37 °C. Optimization experiments revealed that enzyme concentration, pH, temperature, metal ions, and electron acceptors significantly influenced degradation efficiency, defining an operational window suitable for practical applications. LC–MS profiling suggested the presence of transformation products tentatively consistent with oxidative demethylation to aflatoxin P1 (AFP1) and with the formation of AFG2a-like products through subsequent hydration- and oxidation-related transformations. Molecular docking and 100 ns all-atom molecular dynamics (MD) simulations demonstrated stable binding of AFB1 in the T1 copper pocket. Van der Waals and electrostatic interactions, together with a persistent hydrogen bond at Gly323, facilitated single-electron transfer through the intramolecular T2/T3 copper cluster. Principal component and Gibbs free energy analyses confirmed a low-energy, stable conformational ensemble. HepG2 cell assays indicated that BsaMCO-degraded products substantially reduced cytotoxicity and apoptosis compared with native AFB1. Simulated feed experiments further validated enzymatic AFB1 degradation, with approximately 53% reduction after 24 h. Collectively, these findings establish BsaMCO as a safe and effective biocatalyst for AFB1 detoxification, providing mechanistic, structural, and cellular evidence supporting its application in food and feed safety.

## 1. Introduction

Aflatoxins (AFTs) are highly toxic secondary metabolites produced primarily by *Aspergillus flavus* and *Aspergillus parasiticus*, widely contaminating food and feed worldwide [1]. Among them, aflatoxin B1 (AFB1) is the most prevalent and the most potent, exhibiting strong hepatotoxicity, genotoxicity, and carcinogenicity [2]. As an ifuranocoumarin derivative, AFB1 is chemically stable and persistent in food and feed matrices, which amplifies its biological hazards [3]. Exposure to AFB1 can induce liver injury, oxidative stress, and immunosuppression and increase the risk of hepatocellular carcinoma [4,5]. Accordingly, stringent regulatory limits for AFB1 and total aflatoxins have been implemented worldwide. In the European Union, Commission Regulation (EU) 2023/915 establishes commodity-specific maximum levels for AFB1 and total aflatoxins in food. In the United States, the Food and Drug Administration considers most human foods containing total aflatoxins above 20 μg/kg (ppb) to be adulterated and sets action levels of 20–300 μg/kg for animal feed according to species and intended use [6,7,8]. Developing efficient, safe, and application-ready detoxification strategies for AFB1 remains a key priority in food and feed safety.

Current detoxification methods include physical, chemical, and biological approaches. Physical and chemical treatments, such as ozone, ammoniation, and UV irradiation, can reduce AFB1 levels but are often limited by matrix effects, potential nutrient loss, toxic by-product formation, and high industrial costs [9,10]. In contrast, biological strategies, particularly enzyme-mediated degradation, offer high substrate specificity, mild reaction conditions, and the ability to convert AFB1 into less toxic or non-toxic derivatives, making them promising candidates for industrial application [11]. Various microbial enzymes, including laccases, peroxidases, and spore coat proteins, have been reported to degrade AFB1 [12,13]. However, most studies focus on overall toxin removal rather than identifying the specific enzymes responsible, and the practical application of these enzymes is constrained by limited stability under variable pH and temperature, poor activity in complex matrices, and incomplete mechanistic understanding [14,15].

The overall degradation capacity of microbial strains can be influenced by extracellular conditions, enzyme secretion efficiency, and intracellular metabolic coordination [16]. Therefore, a strain exhibiting modest whole-cell degradation does not preclude the presence of highly active intracellular detoxifying enzymes [17]. *Bacillus safensis*, a Gram-positive bacterium with strong environmental adaptability, produces various hydrolytic and oxidative enzymes and has been applied in pollutant degradation and industrial enzyme production [18]. In our laboratory, several Bacillus strains were screened for AFB1-transforming ability, and *Bacillus safensis* Z-1 (GenBank: PX242792.1) was identified as the most promising candidate. Interestingly, although the strain exhibited only moderate overall degradation activity, its intracellular crude enzyme fraction showed significantly higher AFB1-degrading capacity. This finding underscores the value of a reverse enzymology strategy, in which functional detoxifying genes are mined directly from intracellular enzymes rather than inferred from whole-cell performance. Multicopper oxidases (MCOs) are a class of copper-containing redox enzymes that catalyze oxidation of diverse aromatic compounds via their T1/T2/T3 copper centers [19,20]. Given the electron-rich aromatic rings of AFB1, MCOs hold strong potential for its oxidative transformation and are thus prime candidates for AFB1 enzymatic degradation. Nevertheless, current knowledge on MCO-mediated AFB1 degradation remains incomplete, particularly regarding catalytic mechanisms, structural confirmation of degradation products, biosafety evaluation, and enzyme performance in complex matrices, limiting practical deployment [19].

Based on the above background, this study aims to identify and characterize a novel multicopper oxidase from *Bacillus safensis* Z-1 (BsaMCO). To our knowledge, this study is the first to identify a multicopper oxidase from *Bacillus safensis* as an AFB1-degrading enzyme and to combine enzymatic characterization, product profiling, molecular simulation, biosafety evaluation and feed-matrix validation in an integrated framework. Recombinant BsaMCO is obtained through heterologous expression, and its AFB1-degrading activity is systematically evaluated. We further elucidate the molecular mechanism and structural basis for substrate recognition, assess the biosafety of the enzymatic degradation products, and examine the enzyme’s potential application in simulated feed matrices. This work provides a theoretical foundation for molecular engineering, performance optimization, and potential industrial application of BsaMCO, while offering a novel enzymatic candidate and core technical support for controlling AFB1 contamination in food and feed, ultimately promoting practical implementation of enzyme-mediated detoxification strategies in the food and feed industry.

## 2. Materials and Methods

### 2.1. Chemicals and Reagents

AFB1 was purchased from Qingdao Pribio Bioengineering Co., Ltd. (Qingdao, China). For cell-based assays, AFB1 stock solutions were prepared in dimethyl sulfoxide (DMSO), stored protected from light, and freshly diluted with cell culture medium to the desired working concentrations; the final DMSO content in all wells (including vehicle controls) was kept constant and below 0.05% (*v*/*v*). For non-cell-based assays (e.g., in vitro degradation reactions and analytical measurements), AFB1 stock solutions were prepared in methanol and diluted with phosphate-buffered saline (PBS) or the corresponding reaction buffer as required.

For cell culture, Dulbecco’s Modified Eagle Medium (DMEM) was obtained from Wuhan Procell Life Science & Technology Co., Ltd. (Wuhan, China), fetal bovine serum (FBS) was purchased from YiAobang (Beijing, China) Biotechnology Research Co., Ltd. (Beijing, China), and penicillin–streptomycin solution was obtained from Biosharp (Hefei, China). An Annexin V-APC/PI apoptosis detection kit (Kaiji Biotechnology, Nanjing, China) was used for flow-cytometric analysis. For confocal live/dead staining, Calcein-AM and propidium iodide (PI) were purchased from MedChemExpress (MCE, Monmouth Junction, NJ, USA). Unless otherwise stated, all other chemicals were of analytical grade.

### 2.2. Enzyme Production, Expression, and Purification

The coding sequence of BsaMCO was amplified from *Bacillus safensis* genomic DNA and cloned into the pET-28a-SUMO vector (Novagen, Darmstadt, Germany) using BamHI and XhoI restriction sites. The recombinant plasmid was propagated in *Escherichia coli* DH5α, verified by colony PCR and restriction analysis, and then transformed into *Escherichia coli* BL21 (DE3) for protein expression. Transformants were selected on LB agar supplemented with kanamycin (50 μg/mL, Solarbio, Beijing, China).

For recombinant expression, a single BL21 (DE3) colony was inoculated into LB broth containing kanamycin (50 μg/mL) and cultured at 37 °C with shaking until OD600 ≈ 0.6. Protein expression was induced by adding IPTG (Solarbio, Beijing, China) to a final concentration of 0.5 mmol/L, followed by incubation at 25 °C with shaking for 10 h. Cells were harvested by centrifugation at 4 °C, resuspended in pre-chilled PBS containing 1 mmol/L PMSF (Sangon, Shanghai, China), and disrupted by intermittent sonication on ice. The lysate was clarified by centrifugation (12,000 rpm, 30 min, 4 °C), and the soluble fraction was collected for affinity purification.

For purification, the supernatant was loaded onto Ni Sepharose™ 6 Fast Flow resin (Cytiva, Marlborough, MA, USA) pre-equilibrated with PBS and incubated at 4 °C to allow sufficient binding of the His-tagged fusion protein. After removal of the flow-through, the resin was washed sequentially with PBS and PBS containing 10 mM imidazole to eliminate non-specifically bound proteins. SUMO protease (Smart-Lifesciences, Changzhou, China) was then added directly to the resin for on-column cleavage under the recommended conditions. After digestion, the target protein was eluted using a stepwise imidazole gradient (50, 100, 150, 200, 250 and 300 mM), and 5 mL fractions were collected at each concentration. Fractions were analyzed by SDS–PAGE, and those containing BsaMCO were pooled for subsequent experiments. Unless otherwise stated, all procedures were carried out at 4 °C or on ice to preserve enzyme activity.

### 2.3. In Vitro AFB1 Degradation Assay

In vitro degradation of AFB1 was performed using purified, tag-free BsaMCO. For all assays, the enzyme was applied at a concentration of 5 µg/mL. For non-cell-based experiments, AFB1 was dissolved in methanol and added to the reaction system to a final concentration of 2.5 μg/mL. Reactions were initiated by adding the enzyme solution into a PBS-based reaction buffer and incubated at 37 °C for 24 h under dark conditions without shaking. A toxin-only control (AFB1 without enzyme) and an enzyme-only control (enzyme without AFB1) were included to account for non-enzymatic loss and matrix interference.

After incubation, reactions were quenched by adding an equal volume of LC-grade methanol, followed by centrifugation at 12,000 rpm for 30 min to remove proteins and particulates. The supernatant was filtered through a PTFE membrane filter (Biosharp, Hefei, China) prior to chromatographic analysis. Residual AFB1 was quantified by HPLC-UV based on peak area. Chromatographic separation was performed on a ZORBAX SB-C18 column (4.6 × 150 mm, 5 μm, Agilent, Santa Clara, CA, USA) using 50% methanol in water as the mobile phase at 1 mL/min. The detection wavelength was set at 365 nm. The method was validated with a linear range of 0.1–50 μg/mL (R^2^ > 0.998), limit of detection (LOD) of 0.03 μg/mL, limit of quantification (LOQ) of 0.1 μg/mL, and recovery rates of 95–105%. Intra-day precision and inter-day precision were both below 5% RSD, ensuring reliable quantification of AFB1 in the reaction mixtures.

Degradation (%) was calculated as:

Degradation (%) = (Control − Treated)/Control × 100%.

### 2.4. Optimization of Degradation Conditions

To determine the optimal reaction window and evaluate the robustness of BsaMCO-mediated detoxification, degradation conditions were optimized using a one-factor-at-a-time design. Unless otherwise stated, the standard reaction was performed with AFB1 at 2.5 μg/mL (prepared from a methanol stock for non-cell-based assays) and purified tag-free BsaMCO in a PBS-based reaction buffer, followed by incubation for 24 h under dark conditions without shaking. For each condition, a matched toxin-only control (AFB1 without enzyme) and enzyme-only control (enzyme without AFB1) were included to account for non-enzymatic loss and matrix interference. Residual AFB1 was quantified by HPLC-UV.

Substrate concentration. To assess substrate-load tolerance, AFB1 was tested at final concentrations of 2.5, 5, 10, 15, and 20 μg/mL under otherwise identical conditions. The effect of temperature on degradation was evaluated over a broad range of 4–90 °C with 24 h incubation to identify the temperature window supporting maximal degradation. The effect of pH was assessed by adjusting the reaction buffers to pH 2–12 and incubating the enzyme and substrate for 24 h under otherwise identical conditions. To investigate the effects of metal ions on catalytic performance, individual metal ions were added to the standard reaction system at a final concentration of 1 mmol/L, including Fe^2+^, Fe^3+^, Cu^2+^, Co^2+^, Zn^2+^, Mg^2+^, Mn^2+^, Ca^2+^, Ba^2+^, NH_4_^+^, Na^+^, and K^+^. Reactions without added ions served as controls. To examine the influence of redox mediators and electron acceptors, the following compounds were individually supplemented into the standard reaction mixture: SA, PQQ, PMS, ABTS, NAD^+^, NADP^+^, FAD, and DCPIP, followed by incubation for 24 h in the dark prior to HPLC-UV quantification. After incubation in all optimization experiments, reactions were quenched by adding an equal volume of LC-grade methanol, followed by centrifugation (12,000 rpm, 30 min) and filtration through a 0.22 μm organic membrane before chromatographic analysis.

### 2.5. LC–MS Method for Qualitative Profiling

For qualitative profiling, LC–MS analysis was performed on reaction mixtures from the AFB1 control and BsaMCO-treated groups prepared under the standard condition (40 μg/mL AFB1, 37 °C, 24 h). Sample quenching and clarification were performed as described in Section 2.3 and Section 2.4. Clarified supernatants were stored short-term at 4 °C protected from light and analyzed using a Thermo Scientific Q Exactive high-resolution LC–MS system (Thermo Scientific, Waltham, MA, USA). Chromatographic separation was performed on an InertSustain C_18_ column (2.1 × 50 mm, 1.9 μm; Shimadzu, Kyoto, Japan) at a flow rate of 0.2 mL/min. Mobile phase A consisted of water containing 0.1% formic acid, and mobile phase B consisted of acetonitrile containing 0.1% formic acid. The gradient program was as follows: 0–1 min, 90% A; 1–4 min, 90–10% A; 4–8 min, 10% A; 8–8.1 min, 10–90% A; and 8.1–12 min, 90% A.

Mass spectrometric detection was performed in both positive and negative ion modes using a Full MS–ddMS2 acquisition method over an *m*/*z* range of 100–1000. The ion transfer tube temperature was set to 350 °C, the vaporizer temperature to 300 °C, sheath gas pressure to 35 arb, auxiliary gas pressure to 10 arb, and spray voltage to 3.5 kV. Data-dependent MS/MS spectra were acquired for the top five precursor ions using higher-energy collisional dissociation (HCD) with a normalized collision energy of 30. Raw data were viewed and processed using Thermo Xcalibur Qual Browser (version 4.0.27.10, Thermo Fisher Scientific), and feature screening and tentative candidate annotation were further performed using Compound Discoverer (Thermo Fisher Scientific). Putative features were interpreted based on the observed *m*/*z* values, chromatographic behavior, expected molecular formulas, and MS/MS information where available, together with candidate screening results obtained through the Compound Discoverer annotation workflow.

### 2.6. Structural Modeling and Molecular Dynamics Simulations of the BsaMCO–AFB1 Complex

To provide structural insight into the interaction between BsaMCO and AFB1, we employed an integrated computational workflow including structure prediction, molecular docking, and molecular dynamics simulations. Because no experimentally resolved structure of BsaMCO was available, its three-dimensional structure was predicted using AlphaFold3 based on the protein sequence and ligand information [21]. Input features were generated through multiple sequence alignment and template search to capture evolutionary and structural priors. The model underwent iterative optimization of the protein backbone, side-chain conformations, and ligand positioning, while accounting for spatial constraints, geometric complementarity, and intermolecular interaction energies. Model quality was evaluated using the predicted template modeling score (pTM = 0.96), interfacial pTM (ipTM = 0.77), fraction_disordered (0.0), and ranking_score (0.81), indicating a high-confidence structure suitable for downstream analyses.

AFB1 was then docked into the predicted substrate-binding pockets, with particular attention to positioning near the T1 copper center and forming complementary interactions within the hydrophobic cavity. This analysis enabled the identification of key residues potentially involved in substrate recognition and binding.

Molecular dynamics simulations were subsequently performed to evaluate the stability of the BsaMCO–AFB1 complex and to characterize its dynamic interaction pattern in an aqueous environment. For molecular dynamics simulations, protein structures were first optimized by removing redundant ligands, water molecules, and irrelevant ions. Missing atoms and side chains were completed, and atomic types and geometries were checked. Protein topology and coordinates were generated using GROMACS (gmx pdb2gmx) with the AMBER14 force field. The AFB1 ligand topology was built with sobtop using the GAFF force field, and RESP2 charges were calculated in Multiwfn based on ORCA wavefunctions. Ligand geometry was optimized at the B97-3c level. The system was then placed in a periodic cubic box with a 1.0 nm buffer, solvated using the SPC216 water model, and neutralized by adding Na^+^ and Cl^−^ ions.

Energy minimization was performed using the steepest descent and conjugate gradient methods to remove steric clashes, with positional restraints applied to the protein. Pre-equilibration included 100 ps NVT and NPT ensembles at 300 K and 1 bar, respectively, with a V-rescale thermostat and Berendsen barostat, and separate temperature coupling groups for protein and solvent.

The production MD simulation was conducted for 100 ns using the leap-frog integrator. Trajectory and energy data were saved every 10 ps. Hydrogen-containing bond lengths were constrained with LINCS, and long-range electrostatics were treated using PME with a cutoff of 1.0 nm for both Coulomb and van der Waals interactions, including long-range dispersion corrections.

Trajectory analysis and thermodynamic evaluation assessed protein backbone RMSD, per-residue RMSF, radius of gyration (Rg), solvent-accessible surface area (SASA), protein–ligand center-of-mass distance, and hydrogen bond occupancy. MM-PBSA/GBSA calculations decomposed the binding free energy into contributions from van der Waals, electrostatic, and solvation energies, revealing key interface residues.

Finally, principal component analysis and Gibbs free energy landscapes were performed to visualize dominant collective motions and low-energy conformational states of the BsaMCO–AFB1 complex. These analyses collectively provide a structural framework for understanding substrate recognition, complex stability, and the molecular basis of BsaMCO–AFB1 interactions.

### 2.7. Cell Culture

Human hepatocellular carcinoma cells (HepG2-HB-8065, ATCC, Manassas, VA, USA) were maintained in DMEM/F12 complete medium supplemented with 10% fetal bovine serum (FBS) and 1% penicillin–streptomycin at 37 °C in a humidified incubator with 5% CO_2_. Cells were routinely passaged at 70–90% confluence. Briefly, the spent medium was removed and cells were gently washed twice with PBS, followed by trypsinization with 3 mL trypsin at 37 °C for approximately 4 min. Trypsinization was neutralized with complete medium, and the cell suspension was gently pipetted to obtain a single-cell suspension. Cells were collected by centrifugation (1000 rpm, 5 min), resuspended in fresh complete medium, and subcultured at an approximate split ratio of 1:2. Cells in the logarithmic growth phase were used for all experiments.

### 2.8. Cell Treatment Design

To evaluate the biosafety of the enzymatic degradation mixture, HepG2 cells were divided into three groups: (i) vehicle control, without toxin (solvent control matched to treated groups); (ii) AFB1 group, treated with 13.55 μM AFB1, a concentration selected based on preliminary cytotoxicity assays in which the IC50 of AFB1 on HepG2 cells was determined to be approximately 13.55 μM; (iii) degradation-mixture group, treated with the BsaMCO-processed reaction mixture derived from an initial AFB1 equivalent dose of 13.55 μM, in which the AFB1 degradation rate reached ≥50% prior to cell exposure.

For comparability, the same treatment volume was applied across all groups, and the final solvent concentration was kept constant (DMSO < 0.05% *v*/*v*). Prior to cell treatment, the degradation mixture was sterile-filtered to remove potential microbial contamination and particulates. Cells were exposed for 24 h before downstream analyses, including CCK-8 cell viability assay, Annexin V/PI flow cytometry for apoptosis, and CLSM live/dead staining.

### 2.9. CCK-8 Cell Viability Assay

Cell viability was evaluated using a Cell Counting Kit-8 (CCK-8) assay according to the manufacturer’s instructions. HepG2 cells were seeded into 96-well plates and allowed to attach for 18 h prior to treatment. Cells were then exposed to the treatments described in Section 2.8 for 24 h. After treatment, CCK-8 reagent was added to each well (e.g., 10 μL per well) and incubated at 37 °C for an appropriate time (typically 1–2 h) protected from light. Absorbance was measured at 450 nm using a microplate reader. Cell viability was calculated as:

Viability (%) = (OD_treated − OD_blank)/(OD_control − OD_blank) × 100%.

Each condition was tested with at least triplicate wells and experiments were repeated independently.

### 2.10. Flow Cytometry for Apoptosis Analysis (Annexin V-APC/PI)

Apoptosis was assessed using an Annexin V-APC/PI double-staining kit (Kaiji Biotechnology, Nanjing, China). For apoptosis analysis, HepG2 cells were seeded in 6-well plates, allowed to attach for 18 h, and then exposed to the treatments described in Section 2.8 for 24 h. After treatment, cells were harvested using EDTA-free trypsin, collected, and washed twice with PBS (1500 rpm, 3 min). The cell pellet was resuspended in 500 µL binding buffer to obtain a single-cell suspension, followed by addition of 5 µL Annexin V-APC and 5 µL propidium iodide (PI). Samples were incubated for 10 min at room temperature in the dark and analyzed by flow cytometry. Apoptotic populations were quantified using quadrant gating to report live, early apoptotic, late apoptotic, and necrotic cells.

### 2.11. Simulated Feed Degradation Assay

To evaluate the potential application of BsaMCO in a realistic feed matrix, an AFB1-contaminated compound feed sample (2.5 g, 50 μg/kg), obtained from Zhongshi Guoshi Co., Ltd. (Dalian, China), was used in the degradation assay. The feed matrix consisted primarily of corn, soybean meal, corn gluten meal, limestone, calcium hydrogen phosphate, sodium bicarbonate, salt, multivitamin and multimineral premixes, amino acids, and edible oil. The sample was incubated with 10 mL purified BsaMCO under standard reaction conditions (37 °C, 24 h, 200 rpm). The control group received 10 mL of 100 mM imidazole solution to exclude interference from residual elution components. After incubation, AFB1 was extracted and purified from the feed samples using an immunoaffinity column-based procedure according to the manufacturer’s instructions (NCS, Beijing, China). Briefly, 5 g of each feed sample was extracted with 20 mL of 84% acetonitrile in water (*v*/*v*), followed by vigorous shaking or homogenization. The extract was then clarified by centrifugation (6000 rpm, 10 min), and 4 mL of the resulting supernatant/filtrate was diluted with 46 mL of water or PBS before loading onto the immunoaffinity column. After sample loading, the column was washed twice with 10 mL of water. AFB1 was then eluted with 1.5 mL methanol at a flow rate of approximately 1 drop/s, and the eluate was brought to a final volume of 2 mL. The eluate was filtered through a 0.22 μm membrane prior to HPLC analysis. AFB1 degradation efficiency was calculated relative to the control group based on peak area values. AFB1 degradation efficiency was calculated relative to the control group based on peak area values. All experiments were performed in triplicate, and data are expressed as mean ± SD.

### 2.12. Statistical Analysis

All experiments were performed with at least three independent repeats, unless otherwise stated. Data are presented as mean ± standard deviation (SD). Statistical analyses were performed using GraphPad Prism 10.3.0 (San Diego, CA, USA). Comparisons among multiple groups were conducted using one-way analysis of variance (ANOVA) followed by Tukey’s post hoc test, whereas two-group comparisons (when applicable) were analyzed using a two-tailed Student’s *t*-test. A value of *p* < 0.05 was considered statistically significant.

## 3. Results

### 3.1. Cloning and Prokaryotic Verification of BsaMCO

**Figure 1:**
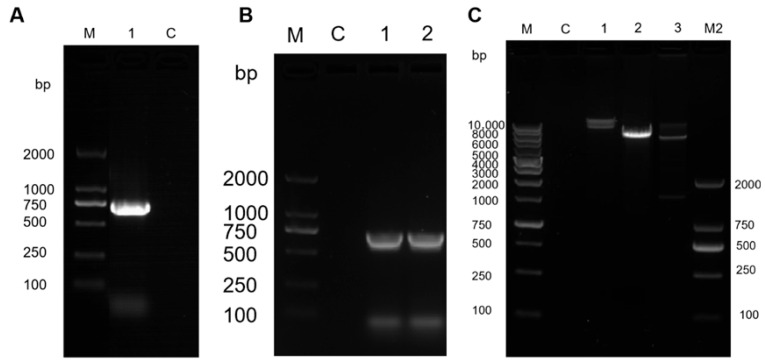
Molecular Verification of the BsaMCO Gene and Recombinant Plasmid. (**A**) PCR amplification of the BsaMCO gene (Lane M: 2 kb DNA marker; Lane 1: BsaMCO gene amplification; Lane 2: negative control); (**B**) Colony PCR verification of BsaMCO-containing transformants (Lane M: 2 kb DNA marker; Lane C: negative control; Lanes 1–2: positive transformant colonies); (**C**) Double digestion verification of recombinant plasmid (Lane M: 10 kb DNA marker; Lane C: negative control; Lane 1: uncut empty vector; Lane 2: empty vector double digestion; Lane 3: BsaMCO transformant double digestion; Lane M2: 2 kb DNA marker).

The BsaMCO coding sequence was successfully amplified, yielding the expected PCR product. Gel electrophoresis showed an amplicon of the expected size, whereas no corresponding band was detected in the negative control (Figure 1A). Successful construction of the recombinant plasmid was supported by colony PCR and restriction digestion analysis (Figure 1B,C). These analyses collectively confirmed the correct insertion and orientation of the BsaMCO coding sequence in the expression vector, providing a foundation for subsequent recombinant expression and purification.

### 3.2. Enzyme Screening, Expression, and Purification

To identify potential AFB1-degrading enzymes from Bacillus species, we first screened several laboratory-isolated *Bacillus* strains for their ability to transform AFB1. Among them, *Bacillus safensis* exhibited the highest activity, with its intracellular fraction (cell lysate supernatant) degrading approximately 48% of 2.5 μg/mL AFB1 after 24 h incubation at 37 °C and 200 rpm. This result indicated that *Bacillus safensis* possesses intrinsic AFB1-transforming capability and therefore warranted further identification of the responsible enzyme.

**Figure 2:**
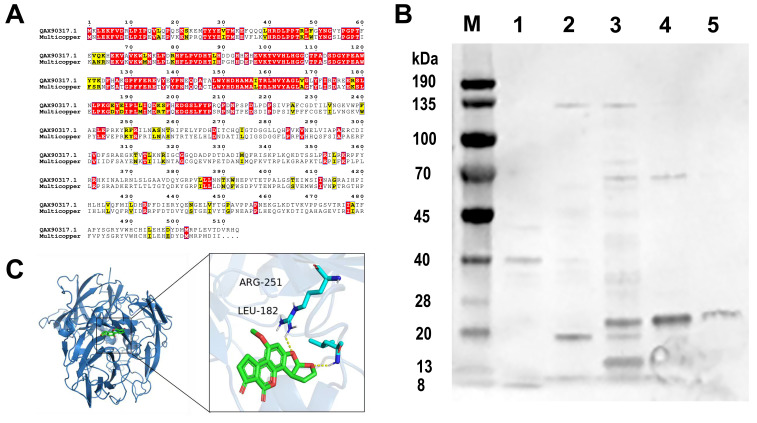
Characterization, Molecular Docking, and Purification of the AFB1-Degrading Enzyme BsaMCO. (**A**) Amino acid sequence alignment of BsaMCO with a reported AFB1-degrading enzyme, showing high identity and extensive coverage (Identical residues are highlighted in red, similar residues are highlighted in yellow, and non-conserved residues are unshaded); (**B**) SDS–PAGE analysis of recombinant BsaMCO purification and SUMO-tag removal (Lane M, protein marker; lane 1, SUMO-protease digestion mixture; lane 2, 10 mM imidazole fraction; lane 3, 50 mM imidazole fraction; lane 4, 100 mM imidazole fraction; lane 5, 150 mM imidazole fraction); (**C**) Molecular docking of AFB1 into the predicted binding pocket of BsaMCO and the corresponding interaction map (The protein is shown as a blue cartoon, the ligand is shown as green sticks, the interacting residues are shown as cyan sticks, and the yellow dashed lines indicate hydrogen-bond interactions).

The BsaMCO candidate was identified by aligning the predicted whole-genome amino acid sequences of *Bacillus safensis* against the amino acid sequence of the reported AFB1-degrading enzyme CotA. A multicopper oxidase family protein was highlighted as the most promising candidate, showing 61% identity, extensive alignment coverage, and an expected value of 0.0 (Figure 2A). Molecular docking further suggested that AFB1 could be accommodated within a predicted binding pocket of BsaMCO, with a favorable binding energy of −7.8 kcal/mol and key interacting residues including Arg251 and Leu182 (Figure 2B).

Recombinant BsaMCO was then expressed in *Escherichia coli* BL21 (DE3) and purified as described in Section 2.2. SDS–PAGE analysis revealed a prominent band at approximately 30 kDa, with the target protein mainly enriched in the 100 mM imidazole fraction (Figure 2C). The purified enzyme was subsequently used for in vitro AFB1 degradation assays and downstream structural and mechanistic analyses.

### 3.3. Effects of Different Conditions on AFB1 Degradation by BsaMCO

**Figure 3:**
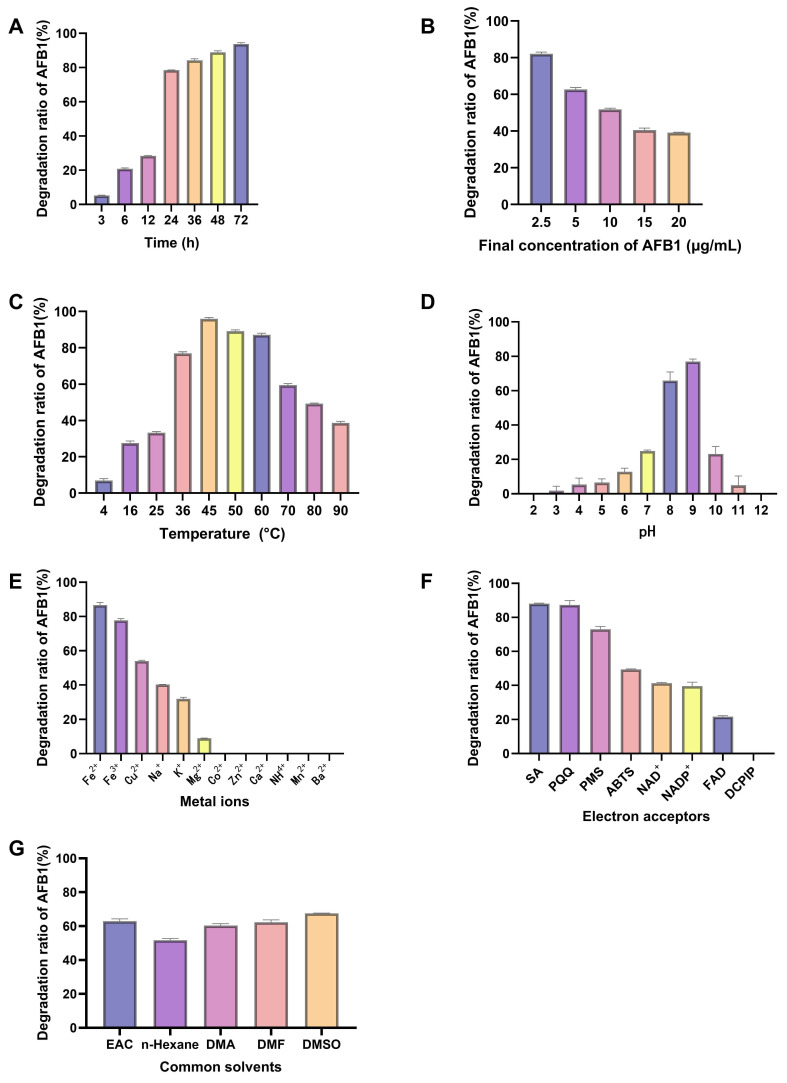
Effects of Different Conditions on AFB1 Degradation by BsaMCO. (**A**) time course over 3–96 h; (**B**) effect of initial AFB1 concentration (2.5–20 μg/mL); (**C**) temperature profile (4–90 °C); (**D**) pH dependence; (**E**) influence of metal ions (1 mM); (**F**) effect of electron acceptors; (**G**) impact of common solvents. Data represent mean ± SD of three independent experiments.

The effects of reaction time, substrate load, temperature, pH, metal ions, electron acceptors, and solvent compatibility on BsaMCO-mediated AFB1 degradation were systematically evaluated (Figure 3). AFB1 degradation increased over time, reaching a plateau of ~78% at 24 h, indicating that this incubation period is sufficient for near-maximal activity (Figure 3A). Substrate load analysis showed robust activity across 2.5–20 μg/mL AFB1, though efficiency decreased at the upper end, suggesting partial saturation of the enzyme active site (Figure 3B).

Temperature and pH strongly influenced BsaMCO activity. The enzyme was most active between 45–60 °C and at slightly alkaline conditions (pH 8–9), whereas extreme temperatures (<25 °C or >80 °C) and highly acidic or alkaline pH substantially impaired degradation (Figure 3C, D). Although maximal BsaMCO activity was observed at 45–60 °C, all subsequent assays were conducted at 37 °C to maintain physiological relevance and ensure compatibility with HepG2 cell-based evaluations. Divalent iron (Fe^2+^) enhanced AFB1 degradation, while other tested ions showed moderate or negligible effects (Figure 3E). Electron acceptors such as SA, PQQ, and PMS improved activity, whereas FAD and DCPIP had little impact (Figure 3F). Common solvents were largely compatible, with ethyl acetate and DMSO having minimal effects, while n-hexane moderately reduced degradation (Figure 3G).

Collectively, these results define a robust operational window for BsaMCO, highlighting its broad thermal and pH tolerance, high substrate specificity, and sensitivity to cofactors and metal ions. The findings provide practical guidance for maximizing AFB1 degradation in vitro and in feed-based applications.

### 3.4. Product Identification by LC–MS

**Figure 4:**
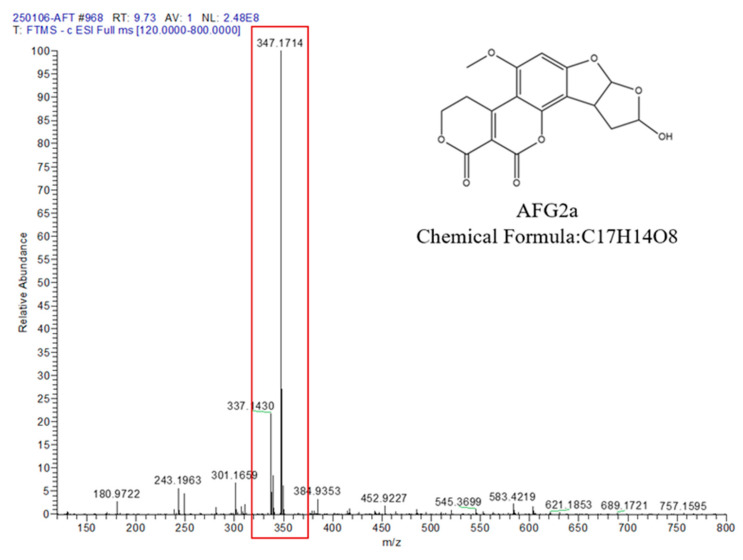
Full-Scan LC–MS Spectrum of the BsaMCO-Treated Reaction Mixture (The major degradation product at *m*/*z* 347.1714; the red box highlights the major degradation product peak; Spectra were acquired in positive electrospray ionization mode).

The enzymatic degradation products of AFB1 by BsaMCO were analyzed using full-scan LC–MS in positive ESI mode. The BsaMCO-treated reaction mixture containing 2.5 µg/mL AFB1 incubated at 37 °C for 24 h was quenched and clarified prior to injection. The LC–MS spectrum detected a major ion at *m*/*z* 347.1714, together with several minor features including a signal at *m*/*z* 299.055 (Figure 4). Based on precursor-ion signals, retention behavior, and expected elemental compositions, these features were tentatively assigned as putative AFB1 transformation products (Table 1). Because no MS/MS fragmentation analysis or authentic reference standards were included in the present study, these assignments should be regarded as provisional rather than structurally confirmed. In particular, the major feature observed at *m*/*z* 347.1714 should therefore be interpreted as a putative AFG2a-like or G2a-type product rather than as definitively identified aflatoxin G2a.

### 3.5. Structural and Dynamic Characterization of the BsaMCO–AFB1 Complex

#### 3.5.1. AlphaFold3-Predicted Structure and Physicochemical Features of the BsaMCO–AFB1 Complex

The three-dimensional structure of BsaMCO was predicted using AlphaFold3, producing a high-confidence model suitable for downstream substrate analysis. Model evaluation metrics indicated excellent reliability, including a predicted template modeling score (pTM) of 0.96, interfacial pTM (ipTM) of 0.77, fraction_disordered of 0.0, and a ranking score of 0.81. The enzyme is composed of three cupredoxin-like domains with well-conserved copper-binding sites, including the T1 mononuclear center and the T2/T3 trinuclear cluster essential for catalysis.

**Figure 5:**
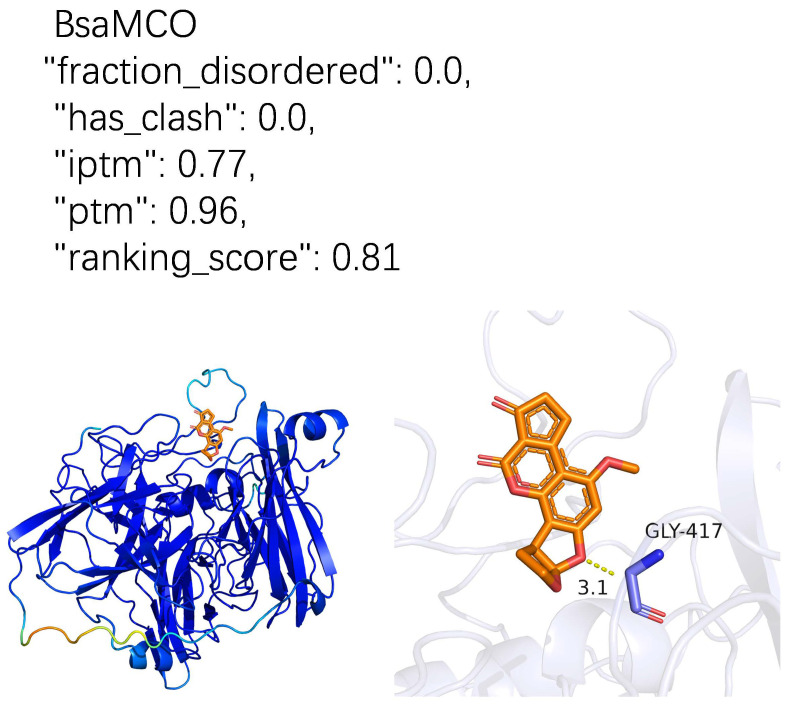
AlphaFold3-predicted BsaMCO–AFB1 Complex. (The model quality is supported by several AlphaFold3 metrics: fraction_disordered reflects the proportion of predicted disordered regions, indicating potential flexible or unstructured areas; has_clash evaluates the presence of steric atomic clashes in the model; pTM measures the confidence of the overall 3D fold; ipTM assesses the predicted quality of intermolecular interfaces; and ranking_score is a composite metric integrating overall structure and interface confidence, used for ranking and selecting among multiple predicted models. In the structure model, BsaMCO is shown as a cartoon colored according to the AlphaFold3 confidence score, with blue indicating higher confidence and yellow/orange indicating lower confidence. AFB1 is shown as orange sticks, GLY-417 as light purple sticks, and the yellow dashed line indicates the interaction distance between the ligand and the residue. This panel illustrates the initial docked complex and its predicted physicochemical environment).

Initial analysis of the BsaMCO–AFB1 complex revealed a clearly defined substrate-binding pocket near the T1 copper site, with a predominantly hydrophobic environment and strategically positioned polar residues, suggesting favorable accommodation for AFB1. The overall surface topology, electrostatic potential, and solvent-accessible volume indicate that the binding cavity is sterically and chemically compatible with the substrate. These features provide insight into potential substrate recognition, orientation, and positioning prior to detailed dynamic simulations (Figure 5).

#### 3.5.2. Molecular Dynamics Assessment of BsaMCO–AFB1 Complex

**Figure 6:**
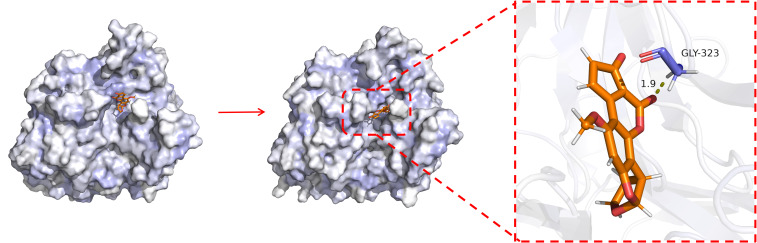
Surface Comparison of the BsaMCO–AFB1 Complex. (BsaMCO is shown as a surface representation in light gray/white, and AFB1 is shown as orange sticks. In the enlarged view, GLY-323 is shown as blue-purple sticks, the yellow dashed line indicates the hydrogen-bond interaction distance between AFB1 and GLY-323).

To evaluate the dynamic behavior and local stability of the BsaMCO–AFB1 complex, a 100 ns all-atom molecular dynamics simulation was performed. The overall conformation of the complex remained largely consistent with the initial docked structure, whereas the binding pocket exhibited subtle rearrangements to accommodate the substrate, in line with an induced-fit mechanism. The hydrophobic cavity deepened slightly, and AFB1 adjusted its position within the pocket, forming a more favorable interaction network. Detailed inspection of molecular contacts revealed a persistent hydrogen bond between Gly323 and AFB1, anchoring the ligand within the pocket and stabilizing its orientation for potential catalytic oxidation. These observations highlight the ability of BsaMCO to rapidly adopt a thermodynamically stable conformation with the substrate securely positioned, providing a structural basis for its enzymatic activity (Figure 6).

#### 3.5.3. Molecular Dynamics Simulation and Thermodynamic Equilibrium of BsaMCO–AFB1 Complex

**Figure 7:**
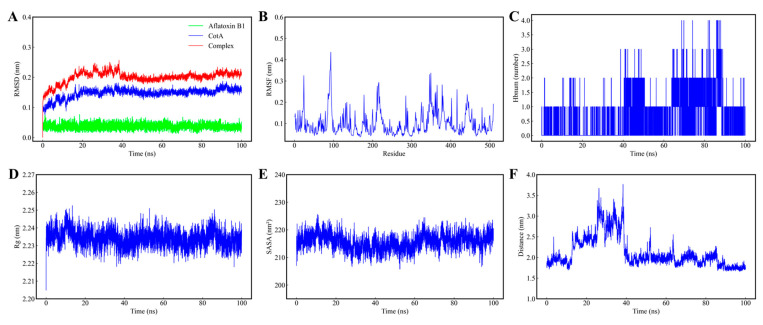
Molecular Dynamics Characterization of the Stability of the BsaMCO–AFB1 Complex. (**A**) RMSD analysis of the protein backbone, free AFB1, and the BsaMCO–AFB1 complex; (**B**) RMSF analysis of BsaMCO residues; (**C**) hydrogen-bond analysis of the BsaMCO–AFB1 complex; (**D**) Rg analysis of the BsaMCO–AFB1 complex; (**E**) SASA analysis of BsaMCO; (**F**) center-of-mass distance analysis of the BsaMCO–AFB1 complex.

The dynamic behavior and thermodynamic stability of the BsaMCO–AFB1 complex were evaluated through a 100 ns all-atom MD simulation. During the initial 40 ns, the system underwent conformational relaxation, side-chain reorientation, and solvent reorganization, consistent with an induced-fit mechanism in which the binding pocket adjusts to accommodate AFB1. After this period, the system reached equilibrium, with the protein backbone (Cα, C, N atoms) RMSD stabilizing at 0.202 ± 0.009 nm, the isolated protein RMSD averaging 0.153 ± 0.008 nm, and the ligand RMSD at 0.036 ± 0.008 nm (Figure 7A), indicating a tightly bound and structurally stable complex.

The Rg remained steady at 2.233 ± 0.004 nm (Figure 7D), and the SASA fluctuated slightly around 215.663 ± 2.733 nm^2^ (Figure 7E), demonstrating that the protein retained overall compactness and stable solvation. The protein–ligand center-of-mass distance initially fluctuated as AFB1 adjusted within the binding pocket and stabilized after 40 ns (Figure 7F), reflecting a persistent binding configuration near the T1 copper center.

RMSF analysis showed that most residues were rigid, while loops at positions 24, 90–93, 346, and 349 exhibited increased flexibility (>0.30 nm), likely facilitating substrate entry (Figure 7B). Hydrogen bond analysis revealed a persistent interaction between Gly323 and AFB1, anchoring the ligand within the pocket (Figure 7C). Collectively, these results suggest that the BsaMCO–AFB1 complex reaches a thermodynamically stable conformation while preserving local flexibility in loop regions to enable substrate access and retention.

#### 3.5.4. Principal Component Analysis and Gibbs Free Energy Landscape of the BsaMCO–AFB1 Complex

**Figure 8:**
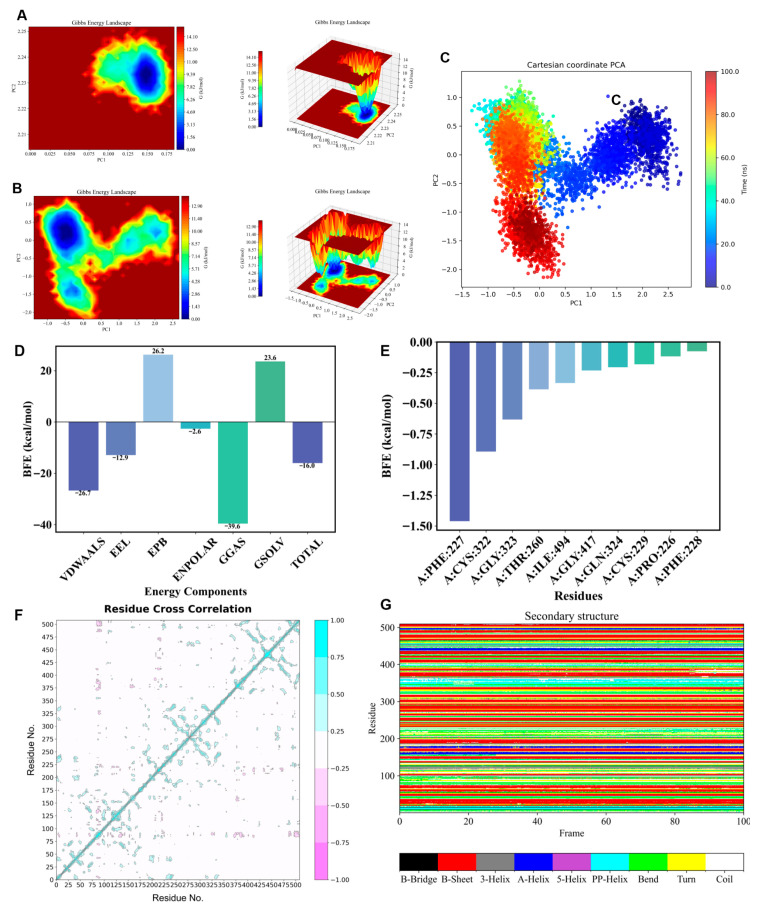
Insights into the Thermodynamic Stability and Atomic-level Interactions of the BsaMCO–AFB1 System. (**A**) RMSD–Rg free energy landscape; (**B**) PCA-based free energy landscape (PC1 vs. PC2); (**C**) PCA scatter plot; (**D**,**E**) MM-PBSA/GBSA binding free energy decomposition (van der Waals (VDWAALS), electrostatic (EEL), polar (EPB), and nonpolar solvation (ENPOLAR) energies); (**F**) Dynamic cross-correlation matrix (DCCM) of Cα atoms; (**G**) DSSP analysis showing temporal evolution of α-helices and β-sheets.

To further investigate the conformational dynamics and energetic stability of the BsaMCO–AFB1 complex, 100 ns molecular dynamics trajectories were analyzed using principal component analysis (PCA), Gibbs free energy landscapes, binding free energy calculations, dynamic cross-correlation matrix (DCCM), and DSSP-based secondary structure analysis. The RMSD–Rg-based Gibbs free energy landscapes (Figure 8A), presented in both two-dimensional and three-dimensional forms, showed well-defined low-energy basins, indicating that the complex predominantly occupied a single thermodynamically stable conformation throughout the simulation. Consistently, the PCA-derived free energy landscape based on PC1 and PC2 (Figure 8B) revealed low-energy regions with only modest spatial extension, suggesting that the complex underwent slight conformational adjustments along the major collective motions while maintaining overall dynamic stability without crossing substantial energy barriers. The PCA scatter plot (Figure 8C) further demonstrated that trajectory points gradually shifted from the initial structure toward these low-energy regions and eventually formed a concentrated cluster, indicating convergence of the system into a stable conformational ensemble after an initial adaptation phase.

Binding free energy analysis further confirmed that formation of the BsaMCO–AFB1 complex was thermodynamically favorable (Figure 8D,E), with a total binding free energy of −16.01 kcal/mol. Van der Waals interactions (VDWAALS, −26.7 kcal/mol) and electrostatic interactions (EEL, −12.9 kcal/mol) were the major favorable contributors, highlighting the importance of both hydrophobic packing and complementary electrostatic interactions at the binding interface. In contrast, polar solvation energy (EPB, +26.25 kcal/mol) partially opposed complex formation, whereas nonpolar solvation energy (ENPOLAR, −2.65 kcal/mol) contributed favorably. Per-residue free energy decomposition further identified several key interface residues that anchor AFB1 within the binding pocket, including hydrophobic and hydrogen-bonding residues that are critical for ligand stabilization.

In addition to energetic stability, analyses of correlated motion and structural integrity supported the functional robustness of the complex. DCCM analysis (Figure 8F) revealed strong correlated motions (Cij > 0.3) among residues surrounding the T1 copper center and along the electron transfer pathway, indicating coordinated movements of functional residues involved in substrate positioning and efficient intramolecular electron transfer. Meanwhile, DSSP analysis (Figure 8G) showed that α-helices and β-sheets remained largely unchanged during the simulation, demonstrating that ligand binding did not perturb the overall protein fold. Together, these results indicate that the BsaMCO–AFB1 complex adopts a stable low-energy conformational ensemble with preserved secondary structure and coordinated internal dynamics, thereby providing a robust structural basis for substrate binding and catalytic activity.

#### 3.5.5. Proposed Single-Electron Transfer Mechanism and Putative Stepwise Transformation of AFB1 by BsaMCO

Based on the LC–MS features, molecular docking, molecular dynamics simulations, and the established catalytic chemistry of multicopper oxidases, we propose a putative degradation pathway for AFB1 catalyzed by BsaMCO. In this model, substrate positioning near the T1 copper center, supported by van der Waals interactions, π–π stacking (Phe227), and a persistent hydrogen bond (Gly323), may facilitate single-electron transfer from AFB1 to the T1 Cu (II) center. One possible early transformation is oxidative demethylation of the methoxy group, yielding a product tentatively consistent with aflatoxin P1 (AFP1).

**Figure 9:**
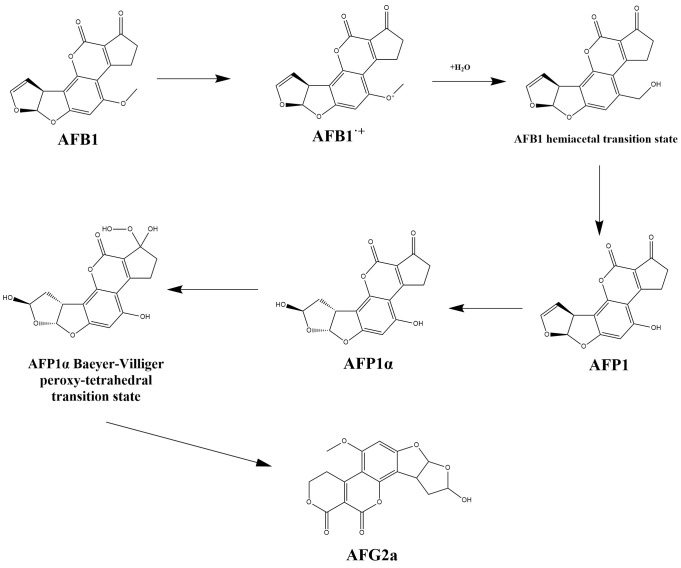
Proposed putative degradation pathway of AFB1 by BsaMCO based on LC–MS features, molecular modeling, and literature-supported multicopper oxidase chemistry.

If AFP1-like intermediates are formed, the pathway may proceed toward products tentatively consistent with AFG2a-like or G2a-type structures through subsequent oxidative and hydration-related transformations. Possible downstream transformations may include hydration of the C8–C9 furan double bond and oxidative rearrangement or lactonization of the five-membered cyclopentenone ring, potentially resembling a Baeyer–Villiger-type process. The newly introduced phenolic hydroxyl in AFP1 enhances its affinity for the T1 copper site, enabling a second single-electron oxidation that generates a phenoxyl radical delocalized across the coumarin and furan rings. Subsequently, the cyclopentenone undergoes oxidative ring expansion, forming a six-membered lactone via oxygen insertion, reminiscent of a Baeyer–Villiger rearrangement. The cumulative effect of methoxy demethylation, double-bond hydration, and ring expansion may yield products with lower toxic potential than native AFB1, although residual toxicity may still remain (Figure 9).

### 3.6. Biosafety Evaluation in HepG2

**Figure 10:**
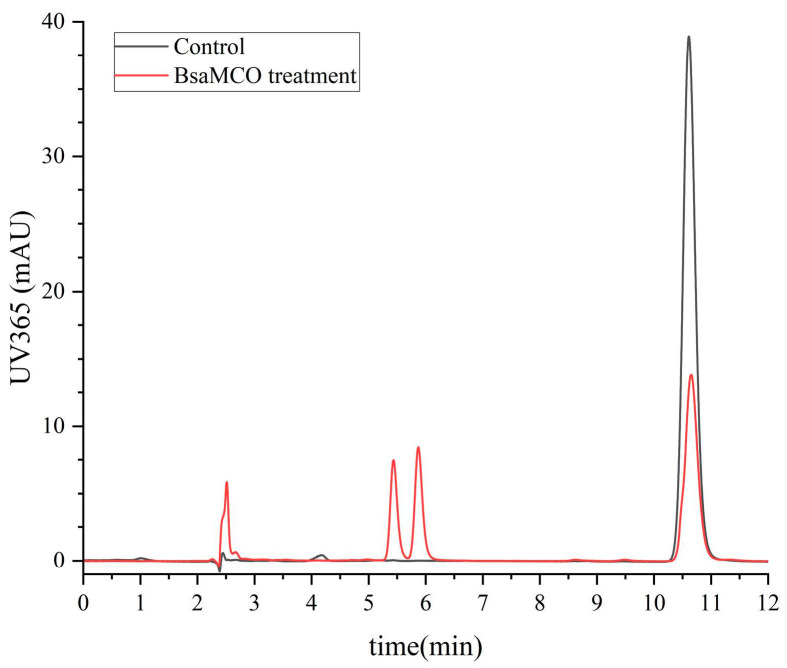
HPLC Analysis of the BsaMCO-Treated AFB1 Reaction Mixture Prior to HepG2 Cytotoxicity Evaluation (Control (black) and enzyme-treated (red) samples are shown; Partial degradation is indicated by decreased parent peak at 10.8 min and new product peaks at 2–6 min).

BsaMCO was first dialyzed to remove small molecules and residual buffer components, and was then incubated with AFB1 at an initial concentration of 20 µg/mL under standard conditions. The resulting mixture was directly applied to HepG2 cell experiments without further concentration in order to preserve enzymatic activity. HPLC analysis of the incubated samples showed partial degradation of AFB1, with new peaks appearing between 2 and 6 min and a reduced intensity of the parent AFB1 peak at 10.8 min (Figure 10), thereby providing samples suitable for cytotoxicity evaluation.

**Figure 11:**
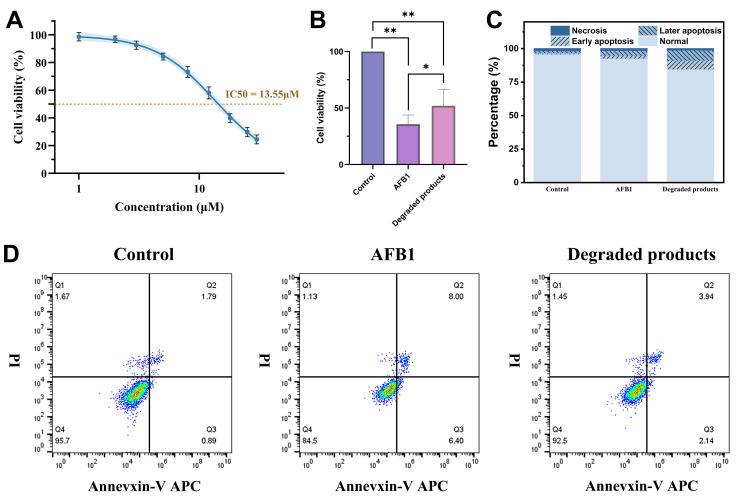
Biosafety Assessment of HepG2 Cells Following Treatment with AFB1 and BsaMCO-Degraded Products. HepG2 cells were treated with 13 μM AFB1 or the BsaMCO-degraded product for 24 h. (**A**) Dose–response curve of AFB1 on HepG2 cell viability determined by CCK-8 assay. Cell viability (%) was calculated relative to the untreated control (mean ± SD, n = 3). The IC50 value (13.55 μM) was estimated from the fitted sigmoidal curve. (**B**) Cell viability after treatment with AFB1 or enzyme-treated degradation mixture. Data represent mean ± SD (n = 3). Significant differences are indicated: * *p* < 0.05, ** *p* < 0.01. (**C**) Flow cytometric analysis of apoptosis using Annexin V/PI staining. Bar graphs show the proportion of viable, early apoptotic, late apoptotic, and necrotic cells. (**D**) Representative flow cytometry dot plots for control, AFB1, and degraded product groups, showing quadrants corresponding to viable (Q4), early apoptotic (Q3), late apoptotic (Q2), and necrotic (Q1) cells. The pseudocolor scale represents cell-event density, with warmer colors indicating higher density and cooler colors indicating lower density.

The biosafety of BsaMCO degradation products was assessed in HepG2 cells by examining cell viability and apoptosis. First, the IC50 of AFB1 in HepG2 cells was determined by CCK-8 assay (Figure 11A), yielding a value of 13.55 μM. Based on this result, an equivalent AFB1 dose (13 μM) was used in subsequent cytotoxicity experiments. As shown in Figure 11B, HepG2 cells treated with AFB1 exhibited a marked reduction in viability compared with the untreated control, with a mean viability of approximately 34%, highlighting the cytotoxic nature of AFB1. In contrast, cells exposed to the BsaMCO-degraded reaction mixture displayed significantly higher viability (~52%) than the AFB1 group (~34%), indicating that enzymatic treatment markedly attenuated AFB1-induced cytotoxicity. However, viability remained well below the untreated control level, demonstrating that residual cytotoxicity persisted after enzymatic treatment under the tested conditions and that detoxification was incomplete. Statistical analysis confirmed that differences between control, AFB1, and degraded product groups were significant (* *p* < 0.05, ** *p* < 0.01).

Apoptotic analysis using Annexin V/PI staining and flow cytometry further corroborated these observations (Figure 11C,D). Total apoptosis, calculated as the sum of early (Q3 quadrant) and late apoptosis (Q2 quadrant), was markedly elevated in the AFB1-treated group relative to control. Specifically, early apoptosis accounted for 6.40% and late apoptosis for 8.00%, while the control group displayed minimal apoptosis (0.89% early, 1.79% late). Cells treated with degraded products exhibited a substantially reduced apoptotic profile (2.14% early, 3.94% late), consistent with partial restoration of cell viability. Necrotic cell populations (Q1 quadrant) were also highest in the AFB1 group and notably lower in the degraded product group, while viable cell populations (Q4 quadrant) were largely preserved following exposure to the enzymatic degradation products. Together, these results demonstrate that BsaMCO-mediated detoxification effectively mitigates the cytotoxic and pro-apoptotic effects of AFB1 in HepG2 cells. Notably, total apoptosis in the degradation-product group (6.08%) remained higher than that in the control group (2.68%), despite being substantially lower than that in the native AFB1 group (14.40%), further indicating that detoxification was incomplete rather than complete under the present experimental conditions.

### 3.7. Simulation Experiment on AFB1 Degradation in Feed by BsaMCO

**Figure 12:**
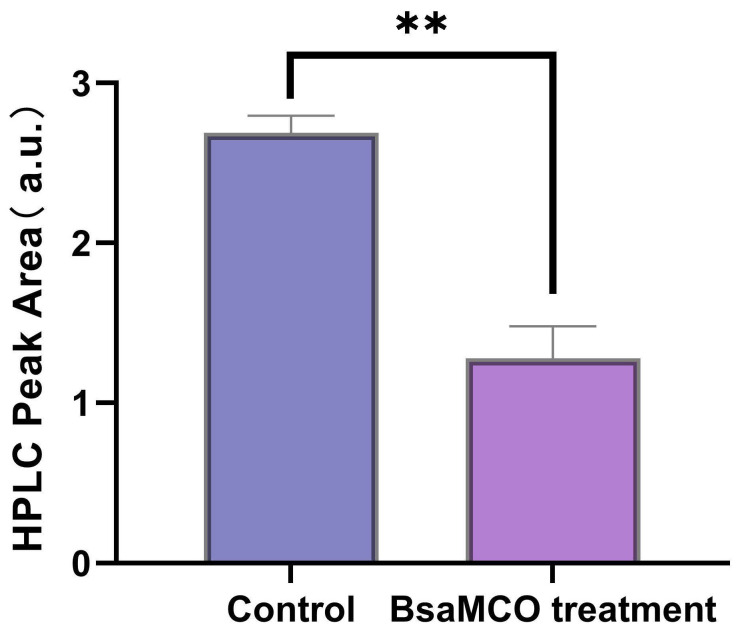
Feed Simulation Degradation of AFB1 by BsaMCO. (Significant differences compared with the control group are indicated as follows: ** *p* < 0.01).

BsaMCO demonstrated substantial AFB1-degrading activity in a feed matrix. (Figure 12) After 24 h incubation at 37 °C, the enzymatic treatment reduced AFB1 content by approximately 53%, as determined by HPLC peak area analysis. The control group, treated with 100 mM imidazole, showed negligible change in AFB1 content, confirming that the observed reduction was attributable to enzymatic activity rather than imidazole. Compared with the in vitro buffer system, the lower degradation efficiency observed in the compound feed matrix likely reflects the greater compositional complexity of the substrate. Components such as corn- and soybean-derived materials, mineral additives, and oil may adsorb AFB1, restrict enzyme accessibility, or partially interfere with catalytic activity. These results demonstrate that BsaMCO retains catalytic activity in a complex feed system, supporting its feasibility for practical application in feed detoxification.

## 4. Discussion

In recent years, the degradation of mycotoxins using microorganisms or their specific enzymes has emerged as one of the most promising industrial detoxification strategies because of its mild reaction conditions, high substrate specificity, and ability to convert toxins into less toxic or non-toxic derivatives [11,17]. However, previous studies have largely focused on whole-cell degradation phenotypes, and such phenotype-driven screening is readily constrained by cell wall permeability, enzyme secretion efficiency, and intracellular metabolic coordination, thereby leading to false-negative results [22]. In this study, a reverse enzymology strategy was employed to identify and heterologously express a novel multicopper oxidase, BsaMCO, from the environmentally adaptable strain *Bacillus safensis* Z-1. Although the wild-type strain exhibited limited whole-cell degradation activity, its intracellular crude enzyme extract and purified recombinant enzyme showed remarkable AFB1-degrading capacity, achieving a degradation rate of over 78% within 24 h at 37 °C without any exogenous mediator. Previous studies have shown that bacterial MCOs generally possess a broad pH tolerance range and good thermal stability, making them suitable for high-temperature processing scenarios such as feed pelleting [17,18]. However, many classical bacterial laccases or MCOs, such as *Escherichia coli* CueO and some wild-type CotA enzymes, exhibit very low direct degradation efficiency toward AFB1 in the absence of exogenous redox mediators, and in some cases are almost inactive [23,24]. For example, StMCO derived from Streptomyces thermocarboxydus achieved a degradation rate of only 31.87% after 24 h in the absence of a mediator [19]. In contrast, BsaMCO achieved a degradation efficiency of over 78% without the need for any mediator, suggesting that the conformation of its substrate-binding pocket and the redox potential of its T1 copper center are better matched to the electron cloud distribution of AFB1, thereby overcoming the limitation imposed by the large steric hindrance of AFB1 and its restricted access to the catalytic center. BsaMCO exhibited an optimal pH in the mildly alkaline range (pH 8–9), a feature that clearly distinguishes it from most laccases, which typically show higher activity under acidic conditions. Fungal laccases commonly undergo hydroxide inhibition under alkaline conditions because OH- binds to the T2/T3 trinuclear copper center [19], whereas BsaMCO retained high activity in an alkaline environment, suggesting that the proton-transfer pathway surrounding its T2/T3 copper cluster may adopt a distinct configuration that resists OH--mediated inhibition. Meanwhile, Fe2+ and electron acceptors such as SA, PQQ and PMS further enhanced its activity, consistent with findings from laccase–mediator systems, in which small-molecule mediators more readily access the T1 copper center, are oxidized into high-energy radicals, and subsequently oxidize the bulkier AFB1 molecule through diffusion [24,25].

The catalytic activity of multicopper oxidases is centred on a conserved copper cluster, comprising the T1 mononuclear copper site located near the substrate-binding pocket and the T2/T3 trinuclear copper cluster responsible for transferring electrons to oxygen and reducing it to water [26]. To elucidate the molecular basis underlying the efficient degradation of AFB1 by BsaMCO, we combined an AlphaFold3 structural model with 100 ns all-atom molecular dynamics simulations to resolve the processes of substrate recognition and dynamic anchoring at atomic resolution. MM-PBSA/GBSA analysis showed that the total binding free energy of the BsaMCO–AFB1 complex was −16.01 kcal/mol, indicating that complex formation was highly thermodynamically favourable; van der Waals interactions (−26.7 kcal/mol) made the dominant contribution, non-polar solvation energy (−2.65 kcal/mol) also favoured binding, whereas polar solvation energy (+26.25 kcal/mol) opposed it. Because AFB1 is highly hydrophobic and rich in aromatic rings [27], its entry into the catalytic pocket is driven primarily by hydrophobic encapsulation by surrounding residues and desolvation effects. This is consistent with previous computational studies of AFB1 binding to laccase and acetylcholinesterase, which showed that polar solvation disfavors the binding of hydrophobic mycotoxins, whereas van der Waals-dominated hydrophobic interactions constitute the principal driving force for complex stabilization [27]. More importantly, the MD trajectories revealed a persistent, high-occupancy hydrogen bond between Gly323 and AFB1, which stably anchors the substrate in a position favourable for electron transfer while restricting its conformational flipping. Previous studies have demonstrated that Gly323 and adjacent loop residues are key determinants of substrate affinity and specificity in several bacterial laccases; in Bacillus subtilis CotA, rational mutagenesis of Gly323, Thr377 and Thr418 markedly altered the hydrogen-bonding network and steric environment, thereby improving catalytic efficiency [18,28]. By contrast, in *Escherichia coli* CueO, AFB1 preferentially binds to the surface Ser243 site distant from the T1 copper centre, resulting in extremely low direct oxidation efficiency [23]. Therefore, the naturally occurring Gly323-centred hydrogen-bonding network in BsaMCO may be a key factor underlying its superiority over enzymes such as CueO. Meanwhile, Phe227 forms π–π stacking interactions with the coumarin ring of AFB1, further stabilizing the planar conformation of the substrate. These multiple non-covalent interactions act synergistically to prolong substrate residence within the pocket, thereby helping to reduce the apparent Km and enhance catalytic efficiency [29].

PCA and free-energy landscapes constructed from RMSD and Rg further revealed the conformational evolution of the BsaMCO–AFB1 complex. During the first 40 ns of simulation, the substrate-binding pocket underwent a slight rearrangement, after which the RMSD stabilized at 0.202 ± 0.009 nm and converged into a single low-energy basin, supporting a typical induced-fit mechanism. RMSF analysis showed that although the overall protein scaffold remained stable, loop regions near residues 24, 90–93, 346 and 349 displayed relatively high flexibility (>0.30 nm). Upon entry of AFB1 into the pocket, these flexible regions underwent adaptive closure, which reduced the influx of bulk water, diminished local dielectric shielding, and further shortened the distance between the substrate and the T1 copper centre. DCCM analysis further showed pronounced coordinated motions between residues surrounding the T1 centre and the electron-transfer pathway (Cij > 0.3), providing a favourable kinetic basis for subsequent single-electron transfer and proton-coupled electron transfer. The potent toxicity of AFB1 arises primarily from two structural motifs: the C8–C9 double bond in the terminal dihydrofuran ring, which is readily epoxidized by CYP1A2 and CYP3A4 to generate the highly electrophilic AFB1-8,9-exo-epoxide, followed by formation of the AFB1-N7-guanine adduct with DNA and the induction of mutagenesis and hepatocellular carcinoma [30,31,32], and the rigid coumarin lactone ring, which is closely associated with acute cytotoxicity, oxidative stress and target-organ injury [18,33]. Therefore, an enzymatic reaction capable of simultaneously modifying these two key toxic structural features is considered an ideal detoxification route. Based on high-resolution LC–MS analysis, we propose that BsaMCO mediates a multistep cascade degradation pathway of AFB1 involving oxidative demethylation, hydration of the furan ring and Baeyer–Villiger-type oxidative lactonization, which is clearly distinct from previous reports in which most *Bacillus* CotA or *Pseudomonas* MCOs convert AFB1 only into AFQ1 or epi-AFQ1 [34,35].

According to the canonical catalytic mechanism of multicopper oxidases, degradation of AFB1 by BsaMCO is initiated at the T1 Cu(II) centre. Through the coordinated positioning effects of Gly323 and Phe227, the electron-rich region of AFB1 is first subjected to electrophilic attack by T1 Cu(II), triggering single-electron transfer to generate an unstable AFB1 cation radical, while T1 Cu(II) is concomitantly reduced to Cu(I) [36,37]. The resulting electron is then transferred through the conserved pathway formed by His419, Cys492 and His497 to the T2/T3 trinuclear copper cluster located approximately 13 Å away, where it is ultimately used to reduce oxygen to water. Following formation of the AFB1 radical, the electrophilicity of its methoxy carbon is increased, allowing a polarized water molecule within the pocket to initiate nucleophilic attack, leading to C–O bond cleavage, rearrangement through a hemiacetal intermediate and loss of one molecule of formaldehyde, thereby yielding the first-generation degradation product AFP1. Although the conventional view holds that O-demethylation of aromatic rings mainly depends on cytochrome P450, quantum chemical and isotope-tracing studies have shown that high-redox-potential multicopper oxidases or laccases are likewise thermodynamically and kinetically capable of catalysing demethylation of aromatic methoxy groups [38]. Replacement of the methoxy group by a phenolic hydroxyl group in AFP1 markedly alters the electronic distribution, steric properties and lipophilicity of the molecule, representing the first step in reducing its intrinsic toxicity [13]. BsaMCO-catalysed turnover did not stop at the AFP1 stage. The newly introduced phenolic hydroxyl group increased the electron-donating capacity of AFP1, making it more susceptible to a second round of single-electron transfer at the active centre, thereby generating a highly delocalized phenoxy radical and further activating the otherwise relatively inert C8–C9 double bond. A water molecule then underwent nucleophilic addition across this double bond, resulting in saturation of the terminal furan ring and formation of a structural core resembling that of AFG2a. Extensive toxicological studies have shown that once the C8–C9 double bond is disrupted, the toxin can no longer be converted in the liver into the carcinogenic exo-epoxide, and its mutagenicity is consequently markedly reduced [39,40]. Nevertheless, this point should be interpreted with caution. Even if the major transformation product is ultimately confirmed to be structurally related to aflatoxin G2a, such a product should not be regarded as non-toxic or fully safe. AFG2a and related derivatives are generally considered less toxic and less carcinogenic than native AFB1 because disruption of the C8–C9 double bond diminishes bioactivation to the highly reactive exo-epoxide; however, residual adverse biological activities may still remain. Therefore, the present results should be interpreted as evidence of reduced toxic potential relative to AFB1 rather than complete toxicological elimination. This distinction is particularly important because the product assignments in the present study remain tentative and were not verified by MS/MS fragmentation analysis or authentic standards. In addition, LC–MS detected a major end product at *m*/*z* 347.1714 (C17H14O8), suggesting that the original cyclopentenone ring underwent a further Baeyer–Villiger-type oxidative lactonization. Classical Baeyer–Villiger oxidation is typically catalysed by FAD- and NADPH-dependent Baeyer–Villiger monooxygenases (BVMOs), as exemplified by the fungal conversion of ZEN into ZOM-1 [37,41]. As a multicopper oxidase that does not require exogenous cofactors, BsaMCO may instead exploit reactive oxygen intermediates generated during the catalytic cycle, or oxygen intermediates activated at the trinuclear copper cluster (TNC), to attack the carbonyl group of the cyclopentenone ring, form a Criegee-like intermediate, and subsequently drive C–C bond cleavage and oxygen insertion [25,26]. Such structural changes would be expected to alter the rigid planar architecture of the fused coumarin-containing system and may reduce interactions associated with the toxicological activity of native AFB1; however, this inference remains mechanistic and does not by itself demonstrate complete loss of toxicity.

Cell-based assays further supported a reduction in biological toxicity following BsaMCO treatment. CCK-8 analysis showed that 13.55 μM native AFB1 reduced HepG2 cell viability to approximately 34%, whereas an equivalent dose of the BsaMCO degradation mixture restored viability to approximately 52% (*p* < 0.01). This result is consistent with previous reports of reduced toxicity in AFP1 and AFG2a-like derivatives: the cytotoxicity of AFP1 is approximately 40% to several-fold lower than that of native AFB1, whereas products that further lose the furan double bond or possess a damaged coumarin ring generally no longer exhibit marked antiproliferative effects [42]. These data indicate that the reduced toxicity of the BsaMCO degradation products arises from at least two aspects: first, hydration of the C8–C9 double bond prevents further CYP450-mediated activation of the products into strongly electrophilic epoxides, thereby eliminating the source of covalent attack on intracellular lipids and nucleic acids [33,43]; second, Baeyer–Villiger lactonization markedly increases molecular polarity and alters the logP value, thereby weakening membrane permeation and entry into intracellular organelles. Flow cytometry results further supported this conclusion from the perspective of cell death mode. The total proportion of early and late apoptotic cells reached 14.4% in the native AFB1-treated group, whereas it decreased to 6.08% in the enzyme-degradation-product group. Although this value remained above that of the blank control, it indicates a substantial attenuation of AFB1-induced apoptosis following BsaMCO treatment. Previous studies have shown that AFB1-induced apoptosis in HepG2 cells is mainly associated with mitochondrial localization followed by ROS burst, disruption of mitochondrial membrane potential, alteration of the Bax/Bcl-2 ratio, promotion of mPTP opening, release of cytochrome c and activation of the p53/Caspase-3 cascade [2,44]. The marked reduction in both early and late apoptosis by the BsaMCO degradation products is attributable to disruption of their key toxic chromophores and reactive sites, which abolishes the capacity to trigger electron transport chain imbalance and lipid peroxidation. Previous studies have also indicated that AFP1 and ring-opened derivatives induce substantially lower levels of ROS after entering HepG2 cells and are less able to markedly perturb p53 expression [42]. In addition, the proportion of PI-single-positive necrotic cells in the degradation-product group also decreased in parallel, indicating that enzymatic treatment likewise attenuated the direct damage of AFB1 to plasma membrane integrity. Overall, this study identified BsaMCO as a bacterial multicopper oxidase capable of efficiently degrading AFB1 and substantially reducing its toxicity without the need for an exogenous mediator, and revealed the molecular basis by which it profoundly dismantles the key toxic scaffold of AFB1 through specific substrate recognition and a multistep oxidative cascade.

From a translational perspective, BsaMCO combines several features that are desirable for practical aflatoxin control, including measurable AFB1 degradation under comparatively mild conditions, retention of activity in a compound feed matrix, and no requirement for exogenous redox mediators. These characteristics are relevant because enzyme-based detoxification is increasingly viewed as a selective and environmentally compatible alternative to physicochemical treatments, whereas many reported multicopper oxidase systems still depend on mediator-assisted catalysis to achieve high efficiency [45]. BsaMCO may therefore warrant further evaluation as a candidate for feed-directed biocatalytic formulations, liquid-phase treatment processes, or immobilized catalytic platforms for contaminated raw materials. Several limitations of the present study should nevertheless be acknowledged. First, the degradation products detected by LC–MS were assigned only provisionally on the basis of precursor-ion signals and expected elemental compositions; more confident structural annotation will require MS/MS fragmentation data and, ideally, comparison with authentic reference standards. This point is particularly important because, even if the major product is structurally related to aflatoxin G2a, such a transformation product cannot be presumed to be toxicologically innocuous, and additional toxicological characterization will be required before any safety claim can be justified. Second, although enzymatic treatment clearly reduced HepG2 cytotoxicity and apoptosis, the residual toxicity was not fully eliminated, indicating that detoxification remained incomplete under the conditions tested. Third, degradation efficiency declined from approximately 78% in buffer to about 53% in the compound feed matrix, suggesting that matrix complexity, toxin adsorption to feed components, or partial inhibition of enzyme activity may constrain performance in more realistic systems. At present, we cannot distinguish whether this reduction is driven primarily by non-specific adsorption of AFB1 to feed components, reduced substrate accessibility and mass transfer, or direct partial inhibition of enzyme activity, because no dedicated matrix-effect experiment was performed in the present study. Finally, biosafety was assessed only in a single in vitro cell model, and the present study did not address long-term enzyme stability, formulation compatibility, process-scale implementation, or performance under industrial operating conditions. These limitations define several priorities for future work. Structural confirmation of the transformation products by LC–MS/MS, together with standard-based verification where available, will be essential for strengthening the mechanistic interpretation. Further studies should also examine enzyme performance across feed matrices of differing composition and under process-relevant variables such as moisture, mixing intensity and temperature. In parallel, enzyme engineering, formulation optimization and immobilization strategies may help improve catalytic efficiency and operational robustness in complex substrates [43]. Broader toxicological evaluation, including additional cell systems and in vivo validation where appropriate, will also be required before the practical use of BsaMCO in food or feed detoxification can be considered with confidence.

## 5. Conclusions

Using a reverse-enzymology approach, we identified and characterized BsaMCO, a multicopper oxidase from *Bacillus safensis*, as an enzyme capable of transforming AFB1 in the absence of exogenous mediators. Enzymatic assays, structural modelling, molecular dynamics simulations, LC–MS profiling, cell-based biosafety analyses, and feed-matrix experiments collectively indicate that BsaMCO can reduce AFB1 levels and attenuate AFB1-associated cytotoxicity. However, these findings should be interpreted with caution. The transformation products were assigned only tentatively, and the major product was not structurally confirmed. Moreover, even if the major product is related to an AFG2a-like derivative, this should not be interpreted as evidence of complete safety, because reduced toxicity does not necessarily imply toxicological innocuity. Consistent with this, residual cytotoxicity remained detectable in the HepG2 model after enzymatic treatment. Therefore, the present data support partial rather than complete detoxification. Further work will be required to confirm product structures, define the toxicological properties of the transformation products, and establish biosafety and practical feasibility under application-relevant conditions.

## Figures and Tables

**Table 1:** Tentative Identification of AFB1 Degradation Products Catalyzed by BsaMCO.

Product	*m*/*z* (ESI)	Retention Time (min)	Mode	Tentative Formula	Relative Abundance (%)
AFB1	313.0701	7.03	+	C17H12O6	100
P1	329.0656	7.25	+	C17H12O7	20–25
P2	331.0807	7.40	+	C17H14O7	10–15
P3	347.0262	7.50	+	C17H14O8	50–60
P4	299.055	7.10	−	C16H10O6	<10

## Data Availability

The original contributions presented in the study are included in the article, further inquiries can be directed to the corresponding author.

## Abbreviations

The following abbreviations are used in this manuscript:

AFB1	Aflatoxin B1
MD	Molecular Dynamics
RMSD	Root Mean Square Deviation
RMSF	Root Mean Square Fluctuation
Rg	Radius of Gyration
SASA	Solvent-Accessible Surface Area
HPLC	High-Performance Liquid Chromatography
